# Soshiho-Tang, a Traditional Herbal Medicine, Alleviates Atopic Dermatitis Symptoms *via* Regulation of Inflammatory Mediators

**DOI:** 10.3389/fphar.2019.00742

**Published:** 2019-07-03

**Authors:** Ji-Hyun Lee, Eun Hee Jo, Bori Lee, Hyeon Min Noh, Sunggu Park, Young-Mi Lee, Dae-Ki Kim, Min Chel Park

**Affiliations:** ^1^Department of Immunology and Institute of Medical Sciences, Medical School, Chonbuk National University, Jeonju, South Korea; ^2^Research Center of Traditional Korean Medicine, Wonkwang University, Iksan, South Korea; ^3^Department of Acupuncture and Moxibustion, College of Korean Medicine, Wonkwang University, Iksan, South Korea; ^4^Department of Oriental Pharmacy, College of Pharmacy and Wonkwang-Oriental Medicines Research Institute, Wonkwang University, Iksan, South Korea; ^5^Korean Traditional Medicine Institute, Wonkwang University, Iksan, South Korea; ^6^Department of Korean Medical Ophthalmology & Otolarynglogy & Dermatology, College of Korean Medicine, Wonkwang University, Iksan, South Korea

**Keywords:** *Soshiho-Tang*, atopic dermatitis, 2,4-dinitrochlorobenzene, keratinocytes, inflammation, oxidative stress

## Abstract

*Soshiho-tang* (SST) is a well-known traditional herbal medicine used for the treatment of many diseases. The aims of this study are to investigate the effects of SST on atopic dermatitis (AD) symptoms and to examine its mechanism. Human keratinocyte (HaCaT) cells were stimulated with tumor necrosis factor alpha (TNF-α)/IFN-γ to induce AD-like keratinocyte environment. 2,4-Dinitrochlorobenzene (DNCB) was used to induce AD-like skin lesions in the dorsal skin of BALB/c mice. SST and dexamethasone were administered orally for 14 day. As a result, SST treatment increased the expression of heme oxygenase-1 (HO-1), an anti-oxidative factor, and the nuclear translocation of NF-E2 p45-related factor 2 (Nrf2). In addition, the treatment also decreased the expression level of inflammatory mediator nuclear factor-κB (NF-κB) and the adhesion molecule intercellular adhesion molecule-1 (ICAM-1). SST treatment (75 and 150 mg/kg) significantly relieved AD symptoms in DNCB-induced AD-like mice by restoring skin thickness, spleen weight, immunoglobulin E (IgE), interleukin 4 (IL-4), pro-inflammatory cytokine expression, and expression of several other mediators. We found that SST alleviates AD-like skin lesions and skin inflammation by modulating various atopic symptoms and inflammatory mediators. Therefore, SST can be used as an alternative drug for the treatment of AD.

## Introduction

Atopic dermatitis (AD), a chronic inflammatory skin disease, is one of the most common skin diseases, characterized by skin hypersensitivity, erythema, itching, relapses, and eczema skin lesions (Leung and Bieber, 2003). The incidence of AD is rapidly increasing worldwide, with about 10–20% of the general population suffering from this disease. Although AD is known to occur due to genetic and environmental factors, abnormal skin barrier function, and immune system abnormalities, the main cause has not been elucidated (Bieber, 2008). Itching is a painful symptom in AD patients and is a major cause of skin barrier dysfunction. Damage to the skin barrier induces the secretion and production of inflammatory cytokines, such as tumor necrosis factor alpha (TNF-α) and interleukin 1-beta (IL-1β) at the site of injury (Wood et al., 1996). It can also induce chronic inflammation and exacerbate the disease by inducing T-helper (Th) 2-mediated inflammation, increasing immune cell penetration, and increasing the production of many inflammatory cytokines (Homey et al., 2006). The promotion of the production of immunoglobulin E (IgE) is one of the characteristics of AD. Therefore, serum total IgE levels can be used to determine the severity in AD patients. In addition, immune cells activated with various cytokines and IgE are known to produce inflammatory cytokines such as IL-4 (Wood et al., 1996).

Chronic inflammation is a well-known common symptom of many diseases. Inflammatory process is initiated by the activation of immune cells, such as mast cells, monocytes, macrophages, and lymphocytes, and followed by the recruitment of these cells into the skin lesions (Ahmed et al., 2017). In the development of inflammatory skin diseases such as AD, leukocyte penetration into the skin is one of the important steps (Gröne, 2002). Increased expression of adhesion molecules such as intercellular adhesion molecule-1 (ICAM-1) on the surface of keratinocytes and vascular endothelial cells induces and increases leukocyte infiltration into inflammatory skin lesions (Dustin et al., 1988). Furthermore, the activation of inflammatory signaling pathways, such as nuclear factor-κB (NF-κB), and several oxidative stress related factors, such as Kelch-like ECH-associated protein 1 (Keap1), NF-E2 p45-related factor 2 (Nrf2), and heme oxygenase-1 (HO-1), are related to the severity of inflammation of AD (Seo et al., 2015; Sun et al., 2015).

Oxidative stress is one of the important factors associated with the onset of atopic dermatitis. The Keap1/Nrf2 signaling pathway has recently been identified as a defense pathway resistant to oxidative stimulation (Yosuke and Dennis, 2016). Nrf2 plays an important role in the expression of antioxidant-related factors that prevent and protect cell damage induced by oxidative stress. Keap1 is well known as an endogenous inhibitor of Nrf2 in the cytoplasm (Motohashi and Yamamoto, 2004). When exposed to oxidative stress, Nrf2 separates from Keap1 and migrates into the nucleus, leading to various antioxidant genes including the HO-1 gene (Ahmed et al., 2017).

As a standard therapy for the treatment of AD, topical or systemic steroids and antihistamines are administered in combination with immunosuppressive agents and antibiotics (Schäkel et al., 2014). However, these long-term remedies are not effective in many AD patients due to severe side effects, such as tolerability, metabolic abnormalities, increased infection, and endocrine abnormalities (Jeziorkowska et al., 2015). Therefore, it is necessary to find new, effective therapies with fewer side effects for the treatment of AD.

Traditional herbal medicine generally has fewer side effects than pharmaceutical drugs and is effective. Medicinal herbs have been used for centuries in Northeast Asia (Korea, China and Japan) to prevent and treat many diseases. In fact, the consumption of these medicinal herbs has been increasing in recent times.

*Soshiho-tang* (SST) is a traditional medicine of Northeast Asia, described in Sang han-ron (150–219 AD in the Chinese Eastern Han Dynasty), an ancient Chinese medicine book. According to Sang han-ron, SST restores heat and dampness in the liver, spleen, and stomach, and thus, SST has traditionally been used to treat chronic liver disease, pulmonary disease, and common colds symptoms such as chills and fever. This medicine, commonly known as *Soshiho-tang* in Korea, is also used in China and Japan, where it is known as “Xiao-chai-hu-tang” and “Sho-saiko-to,” respectively. SST is a traditional herbal prescription formulated from seven herbs including Bupleuri Radix, Pinelliae Tuber, Zingiberis Rhizoma Crudus, Scutellariae Radix, Ginseng Radix, Glycyrrhizae Radix et Rhizoma, and Zizyphi Fructus (**Table 1**). To date, several pharmacological activities of SST have been reported. According to previous experimental studies, this herbal prescription effects treatment of chronic hepatitis (Kusunose et al., 2002) and liver cirrhosis (Chen et al., 2004; Chen et al., 2005) and exhibits a variety of pharmacological properties including anti-inflammatory (Amagaya et al., 1986; Oh et al., 2013), antioxidant (Sakaguchi et al., 1993), anti-tumor (Zhu et al., 2005), anti-obesity (Yoo et al., 2016), anti-asthmatic (Jeon et al., 2015), and immune-regulative activities (Taira et al., 2004; Kang et al., 2009). In addition, SST showed no significant variation between contents depending on the storage period. Long-term administration of SST at a concentration 20 times higher than the human dose was proven to be safe with no toxicity in rats of both genders (Shin et al., 2012; Lee et al., 2013).

**Table 1:** The composition of *Soshiho-tang* (SST).

Herb medicine	Latin name	Family	Source	Ratio (%)
**Bupleuri Radix**	*Bupleurum falcatum* Linne	Umbelliferae	Korea	31.6
**Pinelliae Tuber**	*Pinellia ternate* Breitenbach	Araceae	Korea	10.5
**Zingiberis Rhizoma Crudus**	*Zingiber officinale* Roscoe	Zingiberaceae	Korea	10.5
**Scutellariae Radix**	*Scutellaria baicalensis* Georgi	Labiatae	China	21.1
**Ginseng Radix**	Panax ginseng C.A.Meyer	Araliaceae	Korea	10.5
**Glycyrrhizae Radix et Rhizoma**	*Glycyrrhiza uralensis* Fischer	Leguminosae	China	5.3
**Zizyphi Fructus**	*Zizyphus jujube* Miller var. *inermis* Rehder	Rhamnaceae	Korea	10.5
**Total**				100.0

However, it is not yet known how SST mechanism acts for AD to relieve symptoms. Therefore, in this study, we investigated the effect of SST on AD-like symptoms, such as inflammation and oxidative stress in TNF-α/INF-γ-stimulated human keratinocyte (HaCaT) cells and a mouse model of 2,4-Dinitrochlorobenzene (DNCB)-induced AD-like skin lesions.

## Materials and Methods

### Materials and Reagents

DNCB was purchased from Sigma-Aldrich Co. (St. Louis, Mo., USA). Dulbecco’s modified Eagle’s medium (DMEM), RPMI 1640 medium, fetal bovine serum (FBS), and other tissue culture reagents were purchased from GIBCO BRL (Grand Island, NY, USA). Recombinant human TNF-α and IFN-γ were purchased from ProSpec (ProSpec-Tany TechnoGene, Rehovot, Israel). Specific antibodies for HO-1, ICAM-1, Nrf-2, Keap1, NF-κB p65, Lamine B, β-actin, and corresponding secondary antibodies used for western blot analysis and immunohistochemistry were purchased from Santa Cruz Biotechnology (Santa Cruz, CA, USA). Antibodies against p-IκBα and IκBα were purchased from Cell Signaling Technology (Danvers, MA, USA). Antibody against Loricrin was purchased from Thermo Fisher Scientific (Waltham, MA, USA). Small interfering RNA for HO-1 and negative control was purchased from Bioneer (Daejeon, Korea).

### Plant Materials

SST was prepared from a mixture of minced crude herbs purchased from Omniherb (Korea) and HMAX (China). The identity of each crude herb was established by Professor Je-Hyun Lee at the Korean Medical College of Dongguk University (Gyeongju, Republic of Korea) and Professor Young-Bae Seo at the Korean Medical College of Daejeon University (Daejeon, Republic of Korea). Voucher specimens (2008–KE26–1 ∼ KE26–7) have been deposited at the K-herb Research Center, Korea Institute of Oriental Medicine (KIOM).

### Preparation of SST

Herb components of SST were deposited in the K-herb Research Center, Korea Institute of Oriental Medicine (KIOM). Herbal decoction of SST was made by mixing herbal according to composition (**Table 1**). The aqueous preparation was extracted in distilled water at 100°C for 120 min under pressure (98 kPa) using an electric extractor (COSMOS-660; Kyungseo Machine Co., Incheon, Korea). The extract was filtered through a standard sieve (No. 270, 53 µm; Chung Gye Sang Gong Sa, Seoul, Korea). Afterwards, the filtrate was evaporated and freeze-dried into powder (yield, 22.9%) using a PVT100 freeze-dryer (IlShinBioBase, Yangju, Korea). SST powder was subsequently stored at 4°C until use.

### Ultra Performance Liquid Chromatography (UPLC) Analysis

The UPLC data were obtained using Waters UPLC. The conditions of the UPLC are shown in **Table 2**.

**Table 2:** Ultra performance liquid chromatography (UPLC) conditions of SST.

Instrument	Waters UPLC	
Detector	UV detector	
RP column	Agilent C18 (4.6, 150 mm, 4 µM)	
Column temperature	40°C	
Injection vol	10 µl	
UV wavelength	254 nm, 275 nm	
Mobile phase	A: Water (0.1 vol.% trifluoroacetic acid)	B: Acetonitrile
Time (min)		
0	90	10
5	90	10
15	60	40
25	60	40
26	0	100
30	0	100

### Cell Culture

The HaCaT cell line was purchased from the Korean Cell Line Bank (Seoul, Korea). The HaCaT cells were cultured in DMEM supplemented with 10% FBS, 100 units/ml penicillin, and 10 μg/ml streptomycin (Welgene, Seoul, Korea) and incubated at 37°C in a humidified 5% CO_2_ incubator.

### MTT Assay

Cell viability was confirmed using the 3-(4,5-dimethylthiazole-2-yl)-2,5-biphenyl tetrazolium bromide (MTT) reagent. Cells were seeded at a density of 1 × 10^4^ cells/well in 96-well plates, treated with various concentrations of SST (0, 10, 20, 50, 100, or 500 μg/ml), and incubated at 37°C overnight. Following incubation, MTT solution (5 mg/ml) was added to each well and the cells were further incubated for 4 h. Afterwards, the supernatants were removed, and the crystallized formazan in each well was dissolved in 200 μl dimethyl sulfoxide (DMSO). The absorbance was measured at 570 nm using a microplate reader (Synergy HTX Multi-Mode Reader, BioTek, USA).

### Cell Adhesion Assay

Cell adhesion assay was performed to determine the adhesiveness of THP-1 cells to HaCaT cells. HaCaT cells (7.0 × 10^4^ cells/well) were seeded into 12-well plates. After 24 h incubation, HaCaT cells were pretreated with various SST concentrations (10, 50, or 100 μg/ml) for 30 min and then incubated with TNF-α/IFN-γ (20 ng/ml) for 24 h. THP-1 cells (7.0 × 10^5^ cells/well) were incubated for 30 min in RPMI medium containing 2% FBS and 10 µg/ml 2’,7’-bis-(2-carboxyethyl)-5-(and-6)-carboxyfluorescein, acetoxymethyl ester (BCECF/AM). The fluorescent-labeled THP-1 cells were pelleted and re-suspended in DMEM. Then, these fluorescently labeled THP-1 cells were co-cultured with HaCaT cells at 37°C for 30 min. Afterwards, the non-adherent cells were removed by washing twice with phosphate buffer saline (PBS). The adherent cells were counted using a high-resolution video camera (DXC-960 MD; Sony). Data are expressed as a percentage based on the average number of THP-1 cells in the control group. Each experiment was performed three times.

### Immunofluorescence

Cells were fixed in 4% paraformaldehyde (Sigma-Aldrich, St. Louis, MO, USA) at room temperature in coverslips for 10 min, washed in PBS, incubated in 0.1% Triton X-100, and washed again in PBS. After incubation in 3% bovine serum albumin (BSA), cells were incubated with 1:500 of target protein antibody at 4°C. Next, the plates were carefully rinsed with PBS, stained with Alexa 488-conjugated goat anti-mouse antibody (Invitrogen/Life Technologies, Grand island, NY, USA) for 1 h at room temperature, washed three times with PBS, and incubated with 10 μM 4’,6-diamidino-2-phenylindole dihydrochloride (DAPI) in PBS for 30 min at room temperature. After mounting the coverslip, cells were observed under a confocal microscope (TCS SP5, Leica, Wetzlar, Germany).

### NF-κB p65-DNA Binding Assay

The NF-κB p65-DNA binding was assessed using a NF-κB p65 Transcription Factor Assay Kit (ab133112, Abcam, UK). The nuclear lysate was used to quantify the relative nuclear NF-κB p65-DNA binding. Briefly, a specific double stranded DNA sequence containing the NF-κB responsive binding element was immobilized onto the bottom of wells of a 96-well plate. p65 in the nuclear extract recognized and bound to the NF-κB responsive binding element. p65 was detected using a specific primary antibody against p65, and the signal was enhanced using a secondary antibody conjugated with horseradish peroxidase (HRP). The absorbance was read at 450 nm. NF-κB p65 non-specific competitor dsDNA served as positive and negative controls, respectively. Each experiment was repeated at least three times.

### Small-Interfering RNA (siRNA) and Transient Transfection

Cells were cultured in 6 cm culture plates until reaching about 60% confluence and then treated with 100 nM control siRNA or HO-1 siRNA (sense-CGUAUCCUGGGAUCUCUCU, antisense-AGAGAGAUCCCAGGAUACG) (Bioneer, Daejeon, Korea) in serum-free medium using Lipofectamine RNAiMax transfection reagent (Invitrogen/Life Technologies, Grand Island, NY, USA) for 24 h. Subsequently, the cells were serum-starved for 24 h. The gene-silencing effect of HO-1 siRNA was determined by real-time PCR and western blot analysis.

### Real-Time PCR

HaCaT cells (1 × 10^5^ cells/ml) were seeded into each well in six-well plates. Following a 12 h incubation, cells were pretreated with SST for 30 min and then incubated with TNF-α (20 ng/ml) and IFN-γ (20 ng/ml) for 24 h. Dorsal skin tissues were obtained from all mice after sacrifice. Total RNA was extracted from cells and tissues using 1 ml Trizol solution (Ambion, Austin, TX, USA). After extraction, RNA was quantified using NanoDrop Spectrophotometer (Thermo Fisher Scientific, Waltham, MA, USA). cDNA was obtained using 2 μg RNA and PrimeScript™ II 1st strand cDNA synthesis kit (Takara Bio Inc., Otsu, Japan) according to the manufacturer’s instructions. Real-time PCR was performed using an ABI Real-Time PCR system (Applied Biosystems, Inc., Forster City, CA) with SYBR Green PCR Master Mix (Applied Biosystem, Foster City, CA, USA). Glyceraldehyde 3phosphate dehydrogenase (GAPDH), a housekeeping gene, was used as the internal control. The primer sets are shown in **Table 3**.

**Table 3:** Primers utilized for real-time PCR.

Gene	Forward	Reverse
hHO-1	ATGACACCAAGGACCACAGC	GTGTAAGGACCCATCGGAGA
hFLG	CAGTCAGACTCTAGTACCGCTAAGG	CACTACCATAGCTGCCATGTCTC
hIVL	CCCATCAAAGCAAGAGGAAA	AGCTGCTGATCCCTTTGTGT
hLOR	GTGGGAGCGTCAAGTACTCC	GAGACGCCTCCGTAGCTCTG
mTNF-α	TAGCCAGGAGGGAGAACAGA	TTTTCTGGAGGGAGATGTGG
mIL-1β	GCAACTGTTCCTGAACTCAACT	ATCTTTTGGGGTCCGTCAACT
mIL-6	GACAACCACGGCCTTCCCTA	GGTACTCCAGAAGACCAGAGGA
GAPDH	GTTAGGAAAGCCTGCCGGTG	GCATCACCCGGAGGAGAAATC

HO-1, heme oxygenase-1; FLG, filaggrin; IVL, involucrin; LOR, loricrin; TNF-α, tumor necrosis factor-α; IL-1β, interleukin 1-beta; IL-6, interleukin 6; GAPDH, glyceraldehyde 3phosphate dehydrogenase.

### Western Blot Analysis

Cells were pre-treated with SST for 30 min and incubated with TNF-α/IFN-γ (20 ng/ml) for 24 h at 37°C. The cells were lysed using Pro-Prep protein extraction solution (iNtRON Biotechnology, Seoul, Korea) and centrifuged at 12,000 rpm for 20 min at 4°C, and the supernatants were collected. The extracted proteins were mixed with 2× sodium dodecyl sulfate (SDS)-sample buffer. Lysate of 30 µg of protein was electrophoresed using 10% SDS-polyacrylamide gel for 2 h at 100 V. The separated proteins were transferred to polyvinylidene difluoride (PVDF) membranes (Amersham Pharmacia Biotech). The membranes were blocked with 5% BSA in Tris-buffered saline containing 0.05% Tween 20 (TBST) for 1 h at room temperature and then incubated with the indicated primary antibodies at 4°C overnight. Then, the membrane was incubated with HRP-conjugated secondary antibodies for 1 h at room temperature and visualized using an enhanced chemiluminescence (ECL) western blotting detection reagent (Millipore, MA, USA). Protein imaging was performed using Fusion Fx gel documentation system (Vilber Lourmat, Marne-la-Vallee, France).

### Preparation of Cytosolic and Nuclear Fractions

The cytosolic and nuclear fractions were isolated using NE-PER Nuclear and Cytoplasmic Extraction Reagents (Thermo Fisher Scientific, Waltham, MA, USA) according to the procedure described by the manufacture. The fractions were stored at −80°C before use.

### Animals

Four-week-old male BALB/c mice (21 ± 2 g) were purchased from Samtako Bio Korea (Osan, Korea). The mice were housed in a pathogen-free facility with controlled temperature (22 ± 2°C), humidity (55% ± 5%), and light (12 h light/12 h dark cycle). Animals were provided with sterile standard diet and water *ad libitum*. The protocol for this experiment was approved by the Animal Experiment Ethics Committee of Chonbuk National University (CBNU 2017-0002).

### Induction of AD-Like Skin Lesions in the Mouse Dorsal Skin

Mice were divided randomly into five groups of six mice: 1) Normal group: positive control group, vehicle application; 2) DNCB-induced group: negative control group, DNCB application and oral administration of water; 3) SST 75 group: DNCB application and oral administration of 75 mg/kg SST; 4) SST 150 group: DNCB application and oral administration of 150 mg/kg SST; 5) Dexa group: DNCB application and oral administration of 1 mg/kg dexamethasone. AD-like skin lesions were induced by applying DNCB on the skin. After 1 week of acclimatization, the dorsal skin hair of the mice was removed using an electric razor and hair removal cream. Two hundred microliters of 1% DNCB dissolved in acetone/olive oil (4:1, v/v) was applied to the back of the mice once a day for 3 days. Subsequently, 200 μl of 0.5% DNCB dissolved in acetone/olive oil (4:1, v/v) was applied every 2 days for 14 days. The normal group was applied with only acetone/olive oil (4:1, v/v). The SST groups (75 mg/kg and 150 mg/kg) and Dexa group (1 mg/kg) were orally administered with their respective dose daily from day 4 onwards for 14 days.

### Measurement of Clinical Signs

On the last day of the experiment, the mice were sacrificed and the dorsal skin tissues were isolated. The dorsal skin thickness of each mouse was measured three times using a micrometer (Mitutoyo, Japan). The severity of AD on the dorsal skin tissue of each mouse was assessed visually on the last day of the experiment according to the criteria of severity described previously (Lee et al., 2018).

### Enzyme-Linked Immunosorbent Assay (ELISA)

The mice were anesthetized before sacrificed, and blood was collected. The collected blood was centrifuged at 3,000 rpm for 10 min, and serum samples were obtained. Serum levels of total IgE and IL-4 were measured using ELISA kit (BioLegend, CA, USA), according to the manufacturer’s protocols.

### Hematoxylin and Eosin Staining (H&E)

The dorsal skin tissue specimens were fixed in 10% formalin at room temperature for 24 h and embedded in paraffin wax, and paraffin blocks were serially cut into 6-μm-thick slices. The tissue section was deparaffinized and stained with hematoxylin for 1 min and eosin for 3 min. Then, all tissue slides were dehydrated and sealed with a mounting solution. Histological changes were observed using an optical microscope (Olympus CX21, Olympus America Inc., Melville, NY, USA).

### Immunohistochemistry (IHC)

Specific gene protein expression in mouse dorsal skin tissue was observed using IHC as described previously (Lee et al., 2018).

### Statistical Analysis

All values are expressed as means ± SEM (standard error of the mean). All statistical analyses were performed by using Graph Pad Prism software 5.0 using one-way ANOVA (analysis of variance) to determine the significance of differences between groups. *P* < 0.05 was considered statistically significant.

## Results

### UPLC analysis of SST

**Figure 1:**
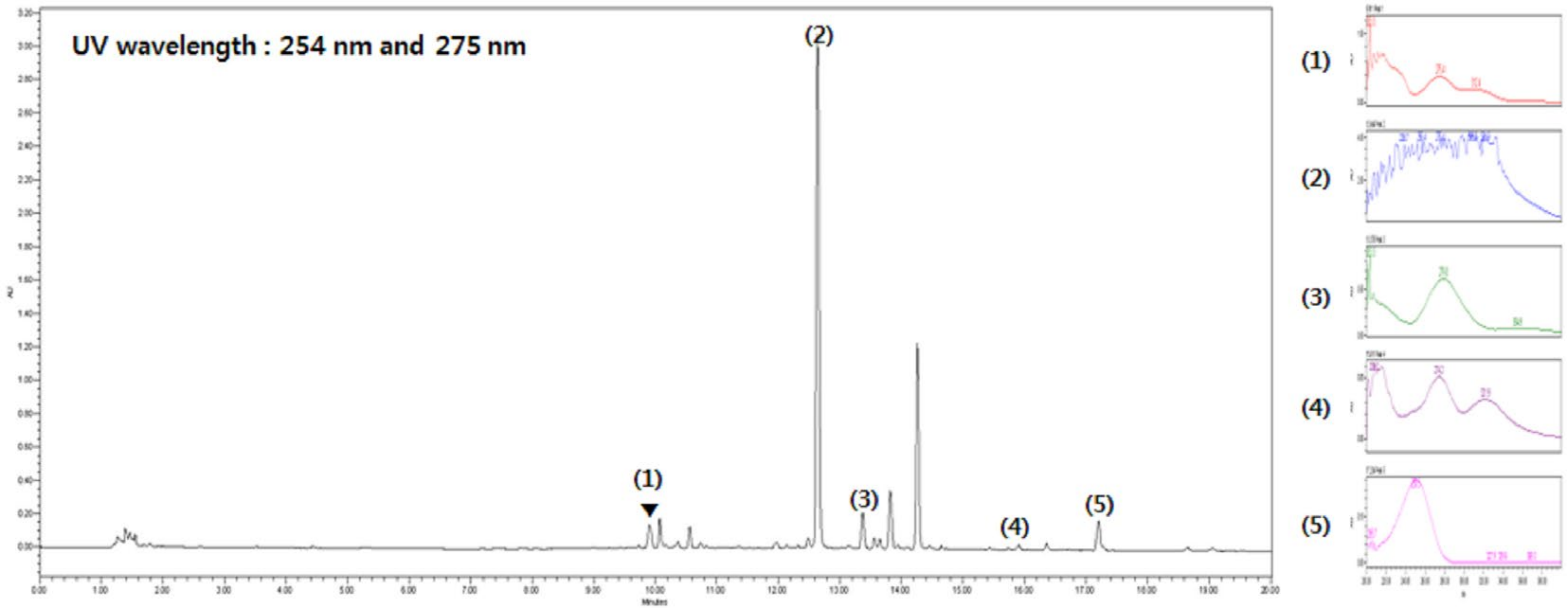
Ultra performance liquid chromatography (UPLC) analysis of *Soshiho-tang* (SST). (1) Liquiritin, (2) Baicalin, (3) Liquiritigenin, (4) Baicalein, (5) Glycyrrhizin.

Five main components of SST were confirmed by UPLC analysis. The UV detector for UPLC analysis was set to 254 nm and 275 nm according to the standard maximum absorption rate. The UPLC analysis of SST water extract is presented in **Figure 1** and **Table 4**.

**Table 4:** Compounds from SST.

	Liquiritin	Baicalin	Liquiritigenin	Baicalein	Glycyrrhizin
Retention time (min)	9.91	12.65	13.38	15.91	17.20
Content (mg/g)	5.44 ± 0.01	0.43 ± 0.01	0.81 ± 0.01	12.8 ± 0.01	0.64 ± 0.01

### Effects of SST on the TNF-α/IFN-γ-Induced ICAM-1 Expression and Subsequent Monocyte Adhesion in HaCaT Cells

**Figure 2:**
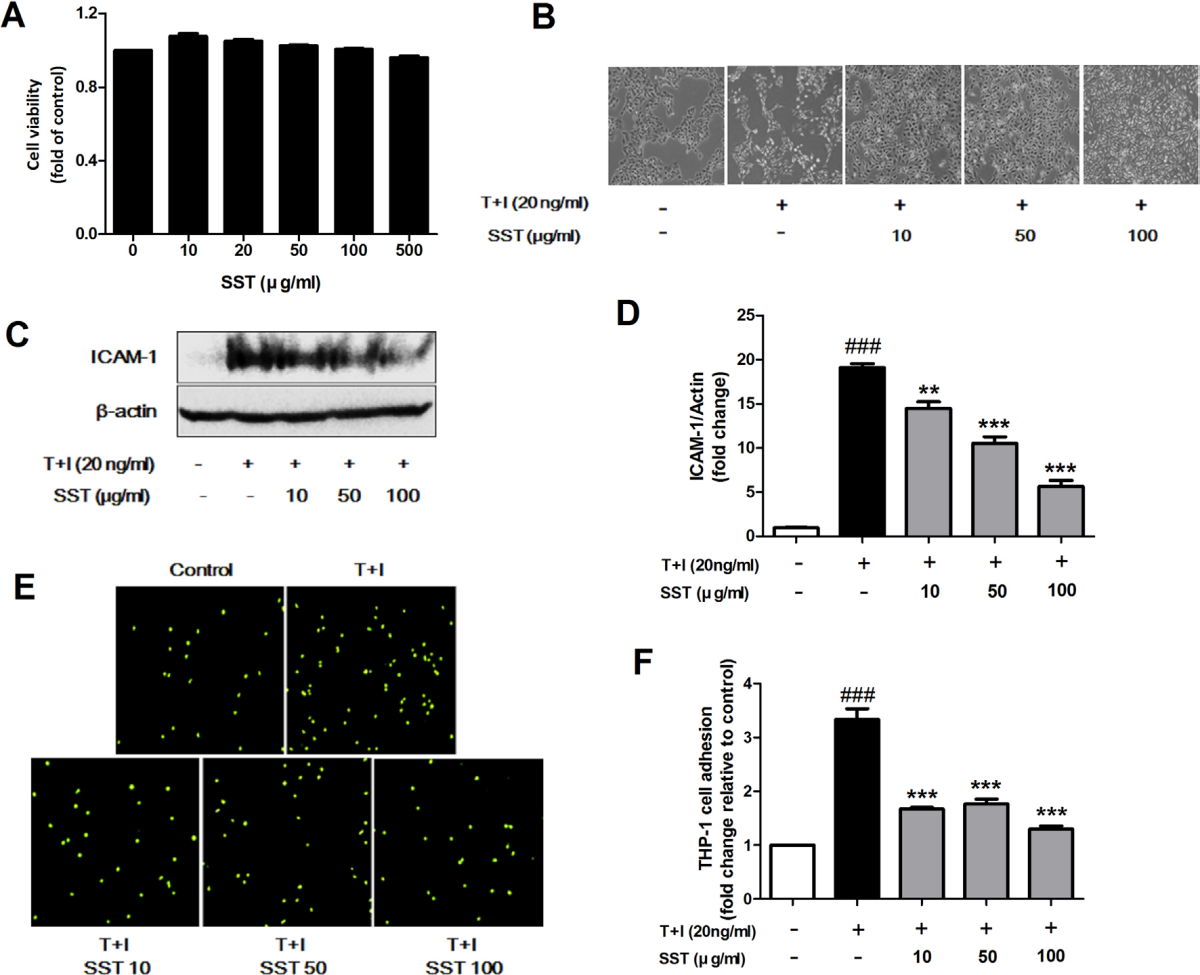
Effects of SST on the tumor necrosis factor alpha (TNF-α)/IFN-γ-induced ICAM-1 expression and subsequent monocyte adhesion in HaCaT cells. Cell viability was analyzed using 3-(4,5-dimethylthiazole-2-yl)-2,5-biphenyl tetrazolium bromide (MTT) assay. Human keratinocyte (HaCaT) cells were incubated in the presence of SST at 10–500 μg/ml or in the absence of SST [Dulbecco’s modified Eagle’s medium (DMEM) only] for 24 h **(A)**. Phase contrast images were taken to observe the morphological changes and proliferation of HaCaT cells due to TNF-α/IFN-γ stimulation and SST pretreatment (magnification: 100×) **(B)**. HaCaT cells were pretreated with various concentrations of SST for 30 min and treated with TNF-α/IFN-γ (20 ng/ml) for 24 h. The protein level of ICAM-1 was determined by western blot **(C, D)**. HaCaT cells were treated with SST and TNF-α/IFN-γ and co-cultured with fluorescent-labeled THP-1 cells at 37°C for 30 min. The adherent cells were counted. The graph is expressed as a fold change of the control **(E, F)**. The data are shown as mean ± SEM of triplicate experiment. ^###^ *p* < 0.001 vs. no-treatment condition, **p < 0.01, ***p < 0.001 vs. only TNF-α/IFN-γ treatment condition.

Inflammatory keratinocytes plays a crucial role in the pathogenesis of AD (Albanesi, 2010). To examine the effect of SST on HaCaT cells, we analyzed the cell’s viability following SST treatment using MTT assay. HaCaT cells maintained high viability at all concentrations of SST within the time period and showed no significant cytotoxicity (**Figure 2A**). Treatment with TNF-α/IFN-γ resulted in dramatic morphological changes in the cells and marked growth inhibition. However, these morphological changes and growth inhibition were restored by SST pretreatment in a dose-dependent manner (**Figure 2B**). In AD, ICAM-1 is known to be involved in the interaction between keratinocytes and inflammatory cells, such as monocytes and macrophages (Dustin et al., 1988; Trefzer et al., 1991). The up-regulation of ICAM-1 increases the adhesion of monocytes to keratinocytes. We investigated the effect of SST on TNF-α/IFN-γ-induced ICAM-1 expression in HaCaT cells and on the subsequent adhesion of monocytes to HaCaT cells. As shown in **Figure 2C and D**, TNF-α/IFN-γ increased the expression level of ICAM-1 in HaCaT cells. However, SST pretreatment markedly inhibited the TNF-α/IFN-γ-induced ICAM-1 in a dose-dependent manner. Consequently, the TNF-α/IFN-γ-mediated induction of ICAM-1 expression increased the binding of THP-1 cells to HaCaT cells, but SST pretreatment suppressed the TNF-α/IFN-γ-induced monocyte adhesion to HaCaT cells (**Figure 2E and F**).

### Effects of SST on Heme Oxygenase-1 (HO-1) Expression and Nrf2 Activation

**Figure 3:**
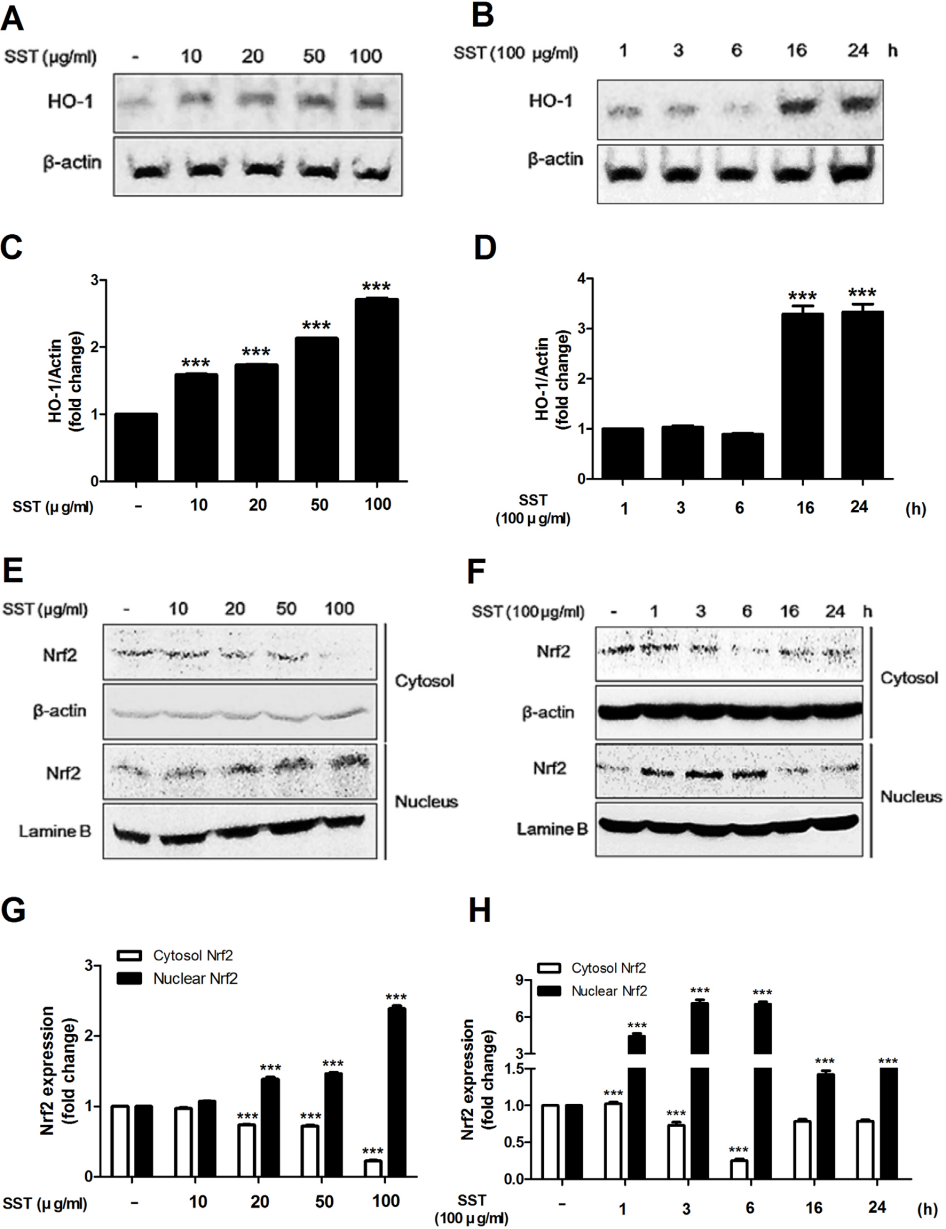
Effects of SST on heme oxygenase-1 (HO-1) expression and Nrf2 activation. HaCaT cells were treated with various concentrations of SST for 24 h **(A, C)** or with 100 μg/ml SST for various lengths of time **(B, D)**. HO-1 protein levels were detected using western blot. HaCaT cells were treated with SST for indicated various concentrations for 24 h **(E, G)** or with 100 μg/ml SST for various lengths of time **(F, H)**. The activations of cytosolic and nuclear Nrf-2 were analyzed using western blotting. The data are shown as mean ± SEM of triplicate experiment. ***p < 0.001 vs. no-treatment condition.

HO-1 is an anti-inflammatory antioxidant enzyme that catalyzes the degradation of heme into ferrous iron, carbon monoxide, and biliverdin (Listopad et al., 2007). HO-1 has been shown to attenuate symptoms in AD-like lesions mice model (Kirino et al., 2008). Recent studies have shown that SST highly increases HO-1 protein expression (Jeon et al., 2015). In the present study, we also observed that SST increased HO-1 protein expression. As shown in **Figure 3A and B**, SST increased HO-1 protein expression in a dose- and time-dependent manner. Following treatment with SST at various concentrations for 24 h, the concentration of HO-1 in the cells was significantly increased at all concentrations. (**Figure 3A and C**). At 100 μg/ml SST treatment, the cells were observed at various time points and reached the maximum expression level at 16 h and persisted for a long time (**Figure 3B and D**). Nrf2 is a transcription factor that regulates the expression of HO-1 (Ahmed et al., 2017). We investigated whether SST affects the expression of Nrf2. SST treatment also considerably increased the nuclear accumulation of Nrf2 and decreased cytosolic Nrf2 in a dose-dependent manner (**Figure 3E and G**). HaCaT cells treated with 100 μg/ml of SST for 1–6 h showed an accumulation of Nrf2 in the nucleus, with corresponding decrease in cytosolic Nrf2 (**Figure 3F and H**). In addition, an SST concentration-dependent decrease in the protein of Keap1 was detected (**Supplementary Figure 1**). These results suggested that SST induced HO-1 expression in HaCaT cells by activating Nrf2.

### Inhibitory Effects of SST on ICAM-1 Expression and Monocyte Adhesion Through HO-1

**Figure 4:**
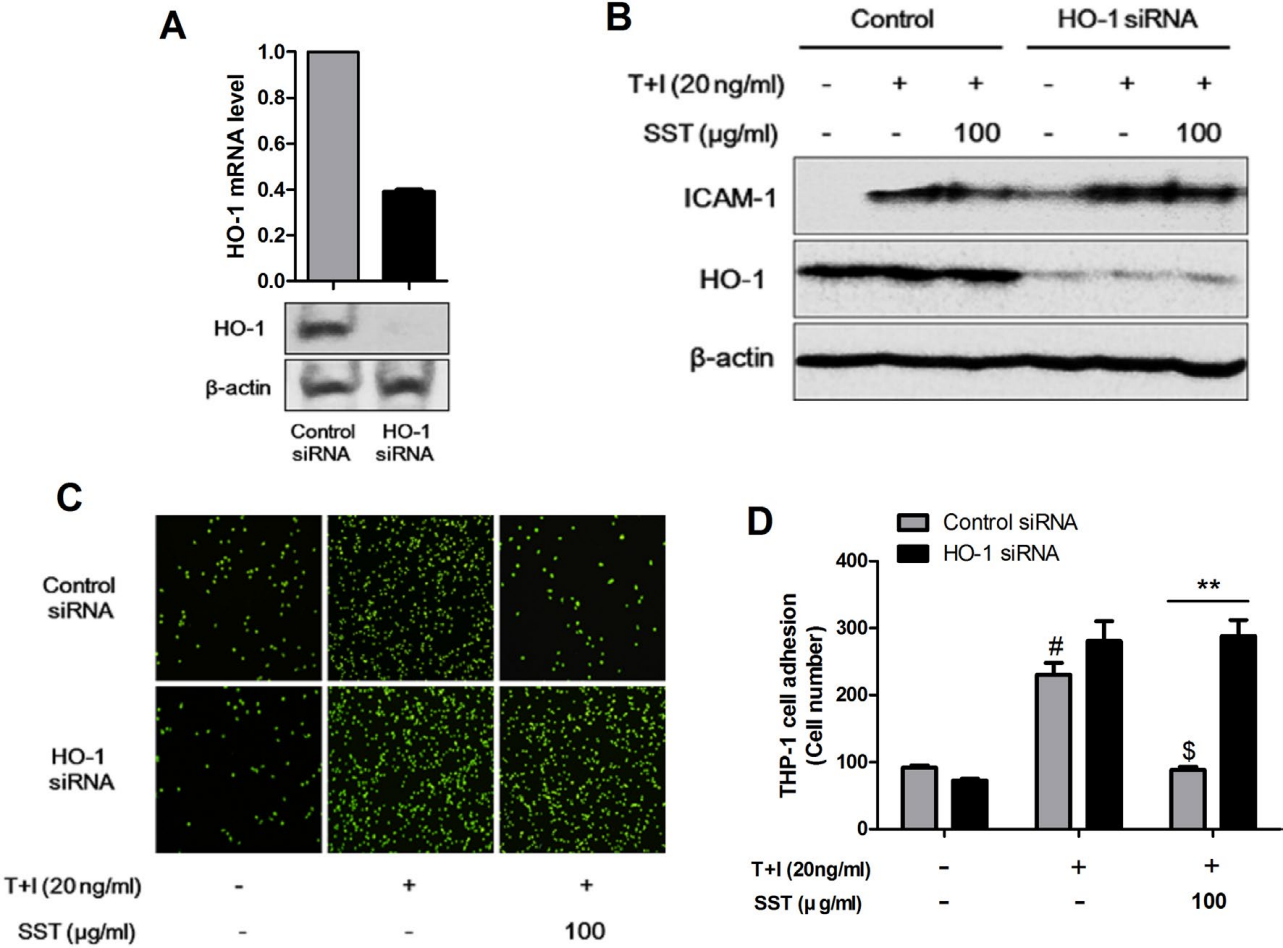
Inhibitory effects of SST on ICAM-1 expression and monocyte adhesion through HO-1. HaCaT cells were transfected with control siRNA and HO-1 siRNA for 48 h. The levels of HO-1 mRNA and protein were confirmed using real-time PCR and western blot **(A)**. HaCaT cells were transfected with control siRNA and HO-1 siRNA and incubated for 48 h. Cells were pretreated with SST for 30 min, followed by stimulation with TNF-α/IFN-γ for 24 h. The protein levels of ICAM-1, HO-1, and β-actin were determined using western blotting **(B)**. Control siRNA- and HO-1 siRNA-transfected HaCaT cells were pretreated with SST for 30 min, followed by treatment with TNF-α/IFN-γ for 24 h. Then, fluorescent-labeled THP-1 cells were added to the HaCaT cells and incubated for 30 min at 37°C. After incubation, the co-cultured cells were washed with PBS to remove the non-adherent cells. The adherent cells were counted, and the graph is expressed as a percentage of the control **(C, D)**. The data are shown as mean ± SEM of triplicate experiment. ^#^ *p* < 0.05 vs. no-treatment condition, ^$^ *p* < 0.05 vs. only TNF-α/IFN-γ treatment condition, **p < 0.01 vs. Control siRNA transfected condition.

We examined the role of HO-1 in TNF-α/IFN-γ-induced ICAM-1 expression in HaCaT cells and in the subsequent adhesion of monocyte to HaCaT cells. In HaCaT cells, HO-1 siRNA transfection reduced HO-1 expression (**Figure 4A**). As shown in **Figure 4B**, the expression level of ICAM-1 was increased in the HO-1 siRNA-transfected HaCaT cells, and the expression level of TNF-α/IFN-γ-induced ICAM-1 was reduced by SST. In addition, the adhesion of TNF-α/IFN-γ-induced monocytes to HaCaT cells was more frequent in HO-1 siRNA-transfected HaCaT cells than in control siRNA-transfected HaCaT cells, and the inhibitory effect of SST on the adhesion was not significantly decreased (**Figure 4C and D**). In conclusion, HO-1 was established as an important mediator in the inhibitory effect of SST on TNF-α/IFN-γ-induced ICAM-1 expression and monocyte adhesion to HaCaT cells.

### Effects of SST on the Nuclear Translocation of NF-κB Induced by TNF-α/IFN-γ

**Figure 5:**
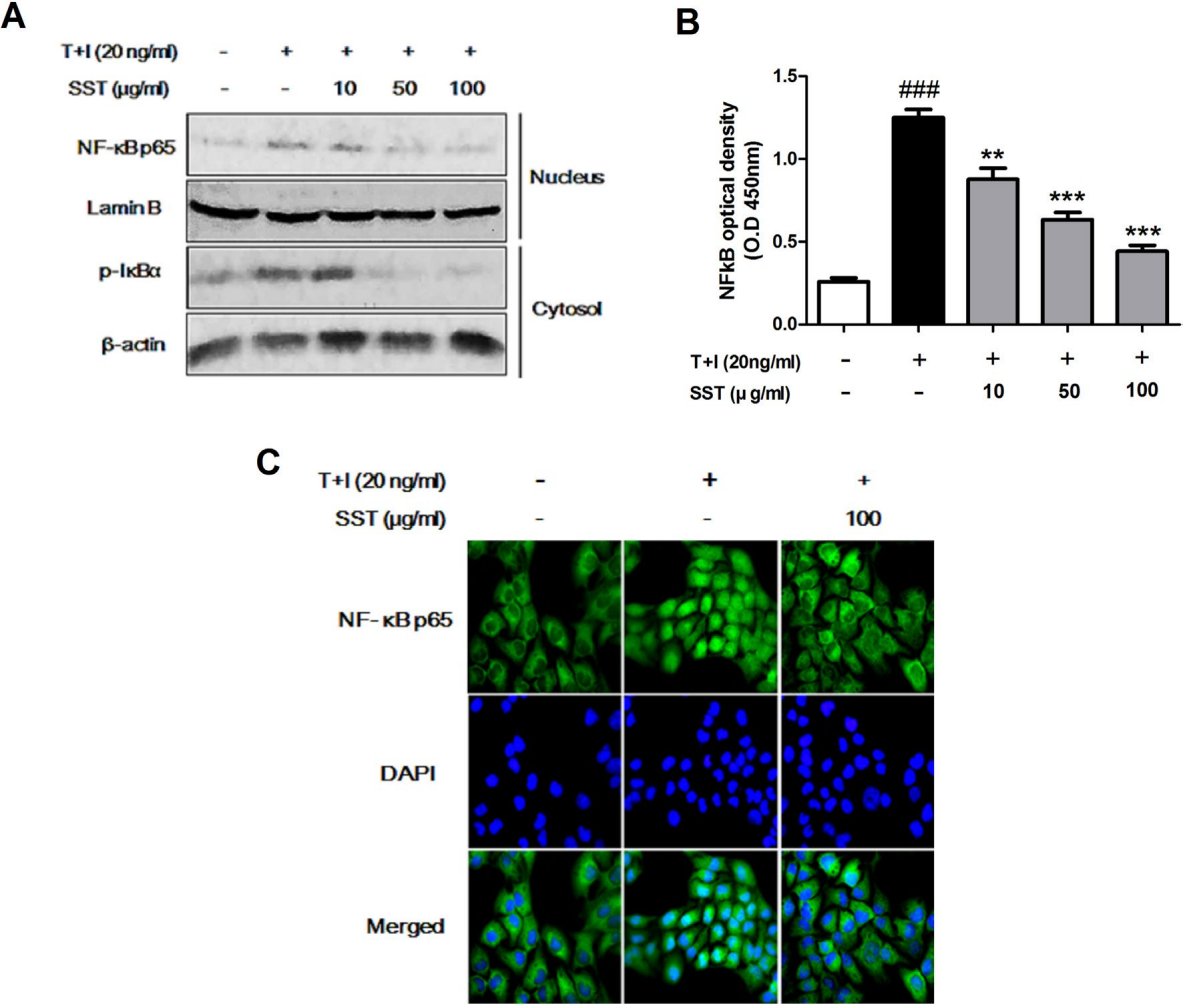
Effects of SST on the nuclear translocation of NF-κB induced by TNF-α/IFN-γ. HaCaT cells were pretreated with the various concentrations of SST for 15 min and then stimulated with TNF-α/IFN-γ for 30 min. The levels of p-IκBα protein in cytosol and NF-κB protein present in the nucleus were examined using western blotting **(A)**. The expression of NF-κB p65 in serum-starved HaCaT cells was observed by immunofluorescence microscopy. Representative images from three independent experiments were displayed **(B)**. Nuclear extracts were analyzed for NF-κB activation by NF-κB transcription factor analysis **(C)**. The data are shown as mean ± SEM of triplicate experiment. ^###^ *p* < 0.001 vs. no-treatment condition, **p < 0.01, or ***p < 0.001 vs. only TNF-α/IFN-γ treatment condition.

Previous studies have shown that TNF-α/IFN-γ stimulation of keratinocytes during AD development induces NF-κB activation that mediates ICAM-1 expression (Kim et al., 2011). The translocation of both NF-κB p65 and p-IκBα in nucleus and cytosolic fractions was increased in the TNF-α/IFN-γ stimulated HaCaT cells, but SST pretreatment markedly decreased the TNF-α/IFN-γ-induced translocation of these proteins (**Figure 5A**). Similarly, NF-κB p65 increased translocation from the cytosol to the nucleus in HaCaT cells stimulated with TNF-α/IFN-γ, but not in SST-pretreated cells (**Figure 5C**). To further confirm this result, we measured NF-κB DNA binding activity to determine whether or not SST inhibited NF-κB activation. **Figure 5B** clearly shows that SST dose-dependently inhibited TNF-α/IFN-γ-induced NF-κB DNA binding activity. These results suggested that SST effectively inhibited TNF-α/IFN-γ-induced NF-κB activation, suggesting a role of NF-κB as a transcriptional activator.

### Recovery Effects of SST on Skin Barrier Proteins in TNF-α/IFN-γ-Treated HaCaT Cells

**Figure 6:**
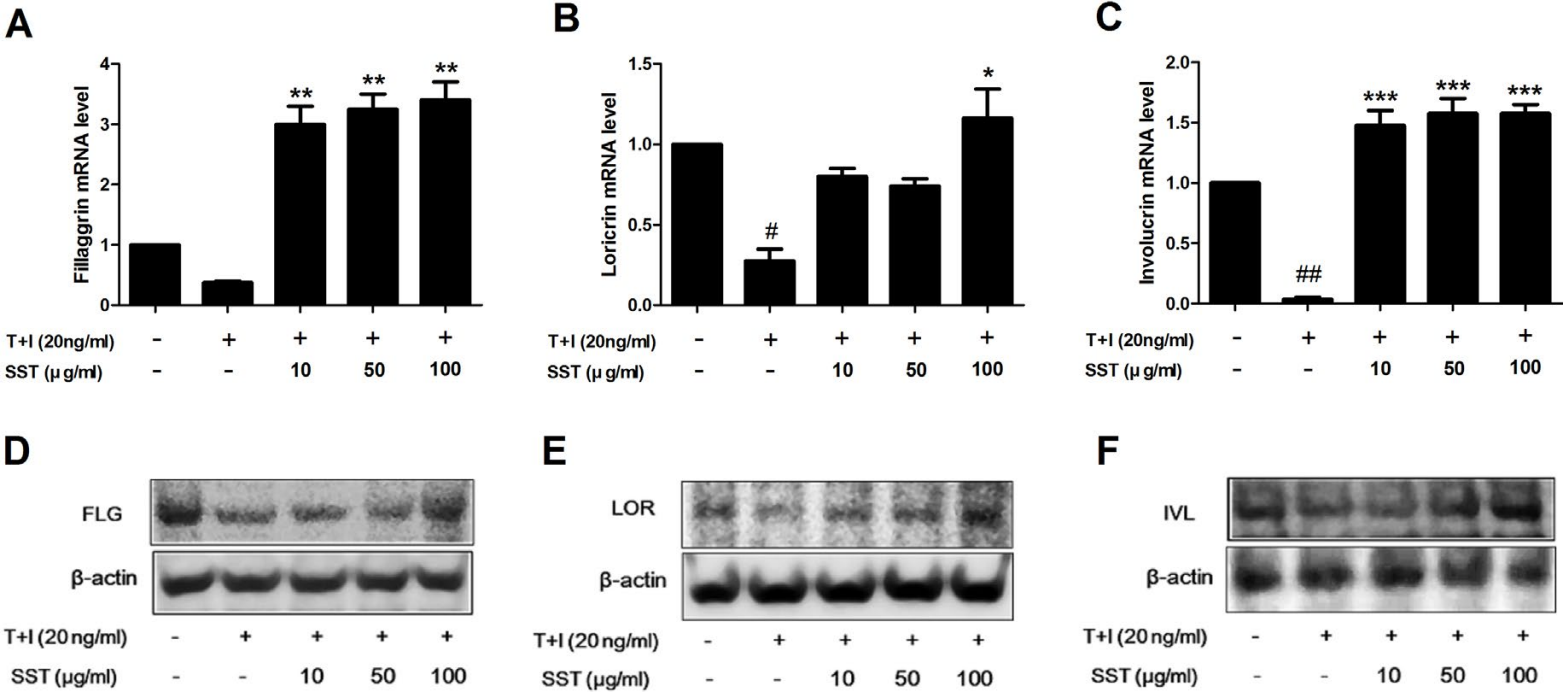
Recovery effects of SST on skin barrier proteins in TNF-α/IFN-γ-treated HaCaT cells. HaCaT cells were pretreated with SST for 30 min and treated with TNF-α/IFN-γ for 24 h. The mRNA levels of filaggrin (FLG) **(A)**, loricrin (LOR) **(B)**, and involucrin (IVL) **(C)** were measured by real-time PCR. Cells were pretreated with SST for 30 min and treated with TNF-α/IFN-γ for 48 h. Protein levels of FIL **(D)**, LOR **(E)**, and IVL **(F)** were measured by western blotting. The data are shown as mean ± SEM of triplicate experiment. ^#^ *p* < 0.05, ^##^ *p* < 0.01 vs. no-treatment condition, *p < 0.05, **p < 0.01, or ***p < 0.001 vs. only TNF-α/IFN-γ treatment condition.

Skin barrier proteins, such as filaggrin (FLG), loricrin (LOR), and involucrin (IVL), have a significant role in the formation of the epidermal skin barrier (Kim et al., 2011). AD is a chronic inflammatory skin disorder characterized by skin barrier damage and reduced skin barrier protein expression. Therefore, we investigated the effect of SST on the expression of skin barrier proteins caused by stimulation of TNF-α/IFN-γ. Stimulation of TNF-α/IFN-γ suppressed the expression of FLG, LOR, and IVL, but both the mRNA and protein expressions of these genes were restored by SST treatment (**Figure 6**). Overall, the results showed that SST played an important role in treating skin barrier dysfunction induced by TNF-α/IFN-γ stimulation.

### Effects of SST on Atopic Dermatitis (AD)-Like Clinical Signs in DNCB-Treated Mice

**Figure 7:**
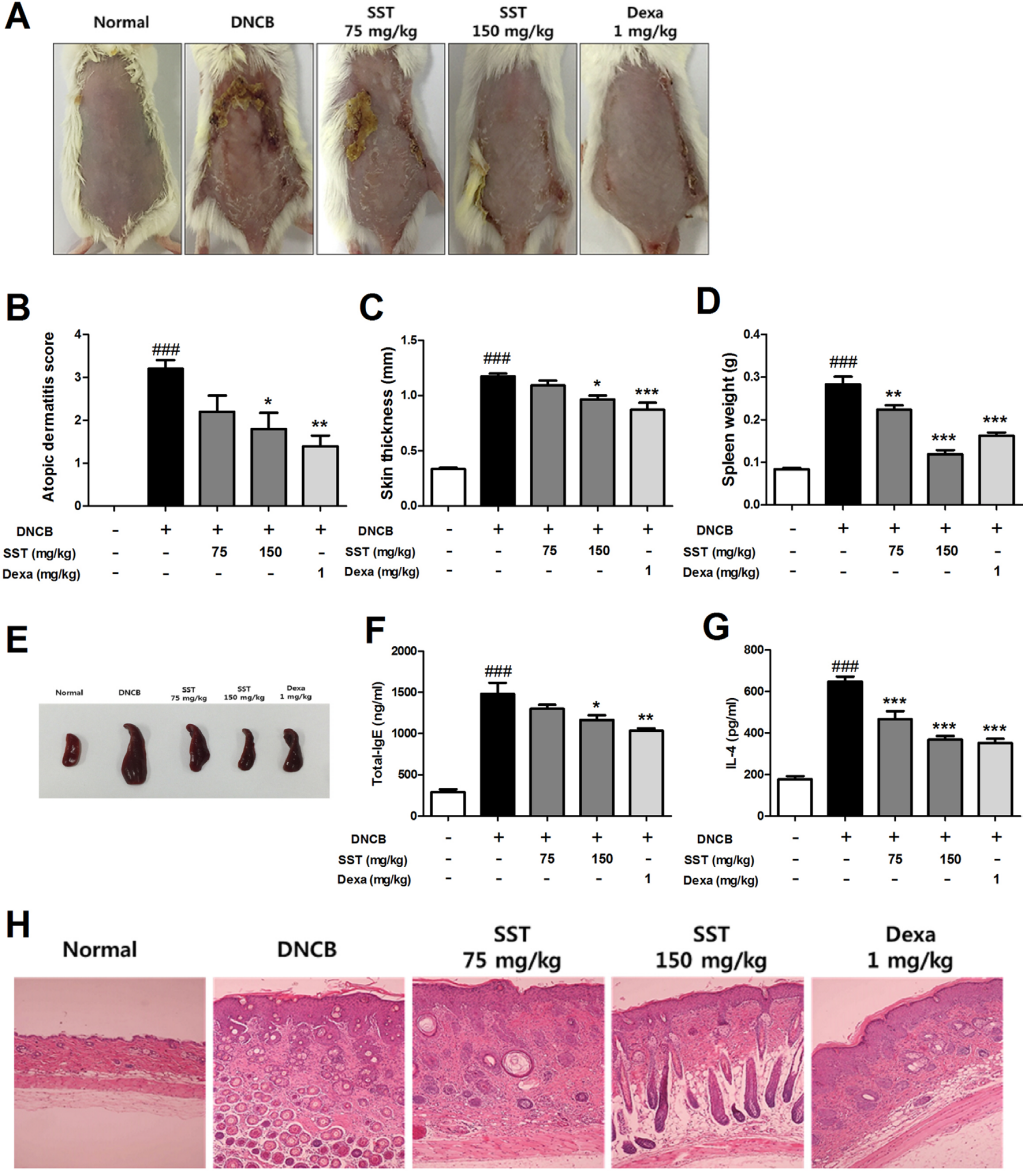
Effects of SST on atopic dermatitis (AD)-like clinical signs in 2,4-Dinitrochlorobenzene (DNCB)-treated mice. To examine the severity of the AD-like lesions, photographs of mouse hairless dorsal skin were taken before mice were sacrificed on the 18th day of the experiment **(A)**. The clinical severity score of all mouse dorsal skin lesions was assessed according to the standard evaluation criteria **(B)**. Dorsal skin thickness of each mouse was measured three times in micrometer **(C)**. The spleen weights of six mice per group were measured electronically **(D)**, and pictures were taken to aid visual comparisons **(E)**. Serum levels of total IgE **(F)** and serum IL-4 **(G)** were measured by enzyme-linked immunosorbent assay (ELISA). The cut dorsal skin was fixed in 10%, cut into 6 μm sections, and stained with hematoxylin and eosin (H&E). Photographs of each group represent images of all mice (*n* = 6). Images were taken at 100× magnification **(H)**. ^###^ *p* < 0.001 vs. Normal group, *p < 0.05, **p < 0.01, or ***p < 0.001 vs. DNCB-induced group.

To analyze the effect of SST on DNCB-induced AD-like skin lesions, we orally administered SST and dexamethasone (as positive control) to the DNCB-induced mice daily for 2 weeks. On the 18th day, the clinical symptoms in the mice were observed. As shown in **Figure 7A**, SST significantly improved AD-like symptoms in the dorsal skin compared to those in DNCB-induced group. In addition, the severity of dorsal skin lesions, which were evaluated with reference to known standards, was significantly reduced in a dose-dependent manner in the SST-treated group (**Figure 7B**). Since DNCB treatment in the dorsal skin tissue caused hyperkeratosis and hypertrophy, the thicknesses of epidermis and dermis were noticeably thicker than those of normal skin. Skin thickness was decreased in a dose-dependent manner by oral administrations of SST and significantly decreased in oral administrations of 150 mg/kg SST group and 1 mg/kg Dexa group (**Figure 7C**). These results were also observed through H&E-staining of the skin tissues (**Figure 7H**). Since SST has been known since the ancient times to play a role in restoring heat and moisture in the liver and spleen, we next investigated the effects of SST on spleen weight in mice. Splenomegaly (spleen hypertrophy) is a symptom of infection and inflammation. If the inflammation is relieved, the size of the spleen can be restored. The spleen weight of DNCB-induced mice was significantly higher than that of the normal mice. The spleen weight of SST-treated mice was dose-dependently decreased compared to that of the DNCB-induced mice (**Figure 7D**). For visual comparison, the spleen representing the average weight was picked and taken for picture (**Figure 7E**). DNCB-induced increase in the serum levels of total IgE and IL-4 was dose-dependently reduced by oral administration of SST (**Figure 7F and G**). Taken together, these results suggested that SST exhibited an excellent effect in alleviating many clinical symptoms of AD-like skin lesions and in relieving inflammation.

### Effects of SST on AD-Related Factors in DNCB-Induced Skin Tissue

**Figure 8:**
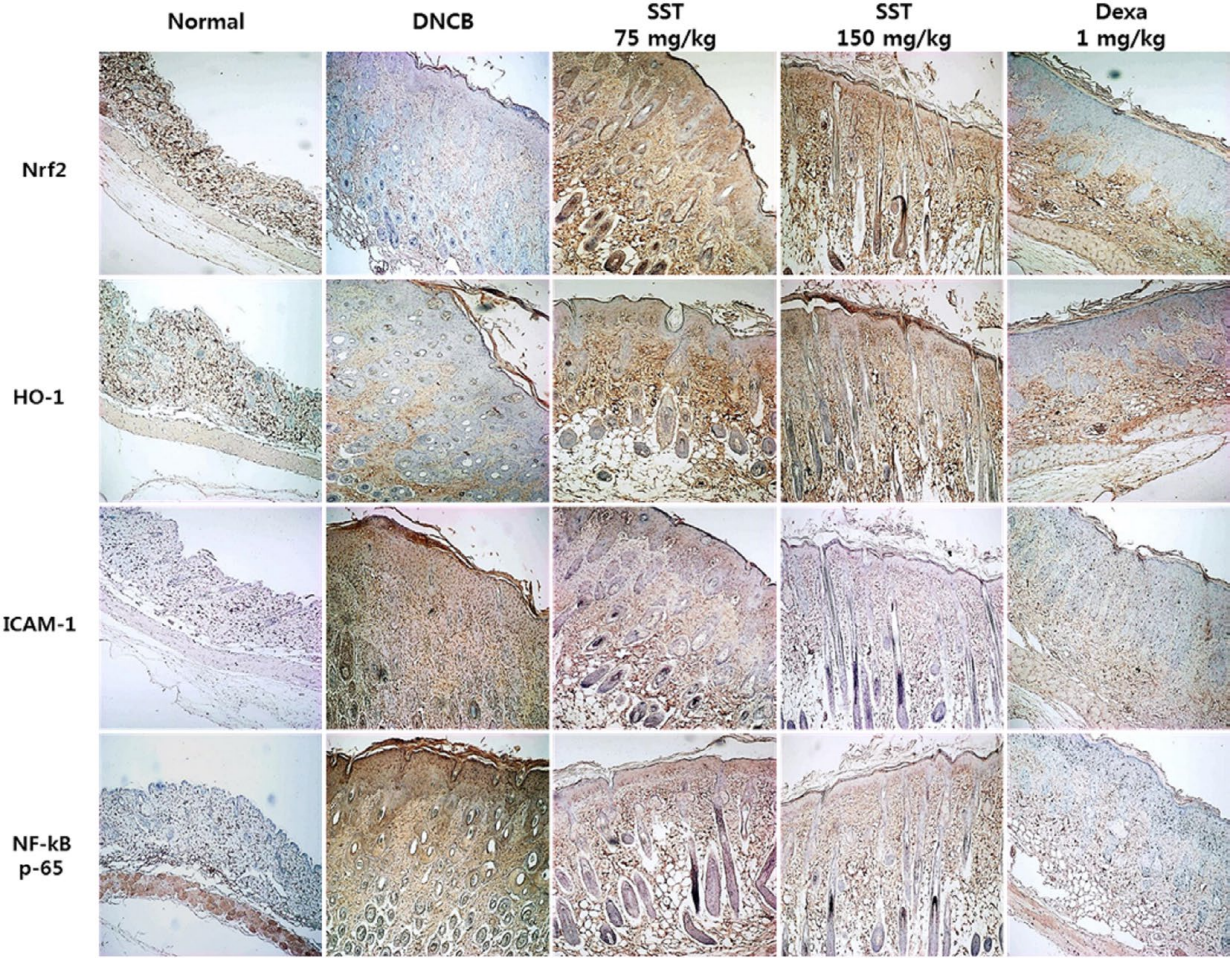
Effects of SST on AD-related factors in DNCB-induced skin tissue. Nrf2, HO-1, ICAM-1, and NF-κB expression levels in the dorsal skin tissue were analyzed by immunohistochemistry (IHC) staining using anti-Nrf2, anti-HO-1, anti-ICAM-1, and anti-NF-κB antibodies. Images were taken at 100× magnification.

Previous studies have shown that increased expression of the Nrf2-dependent downstream factor HO-1 inhibits the expression of ICAM-1 and NF-κB activity (Ahmed et al., 2017). Because the expressions of Nrf2, HO-1, ICAM-1, and NF-κB are related to inflammatory disease, we observed the expressions of indicated factors in the dorsal skin tissue by IHC staining. As shown in **Figure 8**, Nrf2 and HO-1 expressions were significantly decreased, and ICAM-1 and NF-κB expressions were markedly increased in DNCB-induced group compared to those of the normal group. SST considerably increased the levels of Nrf2 and HO-1 and noticeably diminished the levels of ICAM-1 and NF-κB expression in dose-dependent manner, as compared with DNCB-induced group.

### Inhibitory Effects of SST on mRNA Expression Level of Inflammatory Cytokines in DNCB-Induced Dorsal Skin Tissue

**Figure 9:**
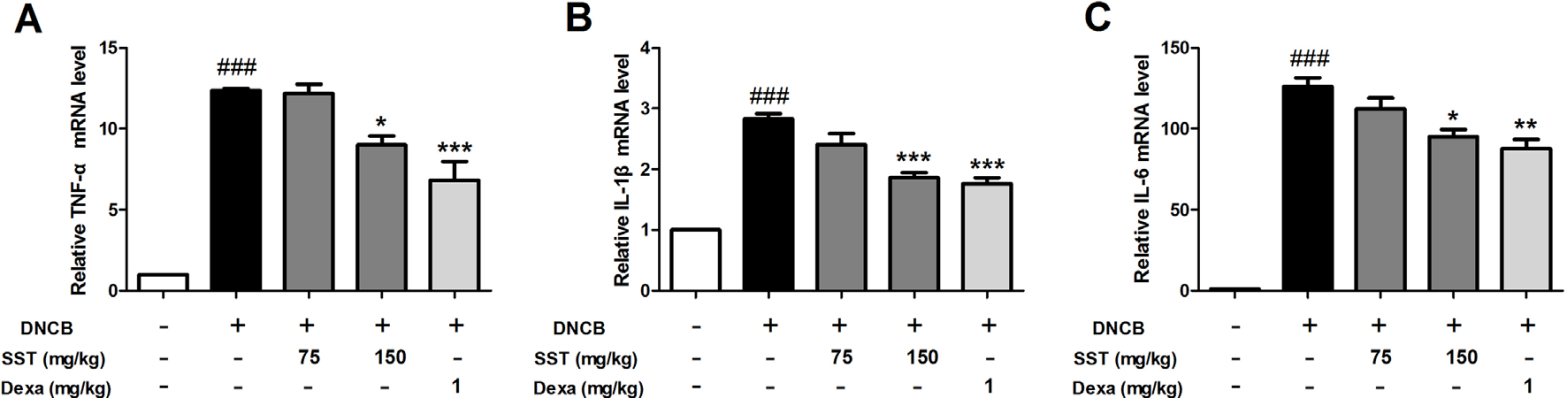
Inhibitory effects of SST on mRNA expression level of inflammatory cytokines in DNCB-induced dorsal skin tissue. The pro-inflammatory cytokine mRNA expression levels of TNF-α **(A)**, IL-1β **(B)**, and IL-6 **(C)** were analyzed using real-time PCR. The data are shown as mean ± SEM of triplicate experiment. ^###^ *p* < 0.001 vs. normal group, *p < 0.05, **p < 0.01, or ***p < 0.001 vs. DNCB-induced group.

To determine the effect of SST on the expression of inflammatory cytokines *in vivo*, the dorsal skin tissues of all mice were collected. Expression of inflammatory cytokines was analyzed using real-time PCR. The levels of pro-inflammatory cytokines TNF-α, IL-1β, and IL-6 were significantly increased in the dorsal skin tissues of DNCB group, compared with those of normal group (**Figure 9**). The levels of pro-inflammatory cytokines were reduced in the SST-treated group (particularly in SST 150 mg/kg group), compared with those of the DNCB group. In addition, the expression of pro-inflammatory cytokines was noticeably diminished in the dexamethasone-treated group. These results suggested that SST suppressed cytokine production in AD-like dorsal skin lesions.

## Discussion

AD is an inflammatory skin disease with recurrence, eczema, itching, and skin hypersensitivity as symptoms. Currently, steroids, antihistamines, and immunosuppressive agents are mainly used to treat AD, but with side effects (Saeki et al., 2016). Therefore, new and effective AD therapies are being developed. So far, plant extracts and derivatives have been shown to modulate the immune function in AD and alleviate AD symptoms (Choi et al., 2014).

Herbal medicines have received widespread attention for decades as an alternative to reduce the side effects of pharmaceutical medicines. Traditional herbal medicine with an herbal formula that has been set for a long time has been used for thousands of years to treat various diseases in Asian countries. Traditional herbal remedies have been used in many diseases, but there is no scientific evidence for their use, so consumers are still concerned about the efficacy and safety of herbal medicines that have not been tested in clinical trials (Cheng et al., 2009). According to previous studies, there was no significant degradation of the content of SST during the storage period (Shin et al., 2012; Lee et al., 2013).

Based on this, we investigated the anti-atopic effect associated with the inflammatory and oxidative stress effects of SST, a safe traditional herbal medicine prescription, and confirmed the possibility of SST as a therapeutic agent in the treatment of AD.

Keratinocytes form the stratum corneum and act as a first defense against external stimuli. Therefore, epidermal keratinocyte dysfunction is an important cause of AD pathogenesis. The keratinocytes of the skin of the AD are deformed, and then the function of the protective membrane is weakened. Also, as inflammation occurs repeatedly, the skin becomes thicker and the itching becomes worse. If the skin is scratched by itching, various pro-inflammatory cytokines and chemokines are produced continuously in the keratinocytes. In inflammatory skin diseases such as AD, various cytokines, including TNF-α and IFN-γ, and stressors increase the expression of adhesion molecules in keratinocytes and induce leukocyte infiltration into inflamed skin lesions (Dustin et al., 1988). One of adherent molecules, ICAM-1, mediates the interaction between keratinocytes and immune cells, which is one of the major roles in the pathophysiology of AD.

Oxidative stress is also known to be one of the causes of AD (Ji and Li, 2016). Antioxidant enzymes play a crucial role in protecting cells from the effects of oxidative stress. Thus, the improvement of the antioxidant defense system is an important strategy for the prevention of AD.

Nrf2, a transcription factor, separates from Keap1 in the cytoplasm and translocates from the cytosol to the nucleus and binds to the ARE, an antioxidant response element in the nucleus, to upregulate the expression of antioxidant enzymes, including HO-1, and induce cell protection (Ahmed et al., 2017). In addition, Nrf2 is involved in the recruitment of inflammatory cells and is known to contribute to anti-inflammatory processes (Braun et al., 2002; Chen et al., 2006).

The induction of HO-1 expression is one of the important mechanisms to protect cells from oxidative damage. By-products produced by HO-1 are known to exhibit cytoprotective, anti-inflammatory, and anti-oxidative effects (Ryter et al., 2006). Recently, HO-1 expression has been shown to modulate the inflammatory response in AD patients with increased oxidative damage-induced inflammation.

NF-κB is a protein complex found in most cells that acts as a mediator in various processes, such as immune response, inflammation, apoptosis, cell growth, and development. It is an inflammatory mediator activated by external stimuli or oxidative stress, and it induces the transcription of pro-inflammatory cytokines, such as IL-6, TNF-α, and IL-1β (Barnes and Karin, 1997). Inflammation stress further activates NF-κB and produces more cytokines. The activation of the Nrf2 system plays a role in inhibiting the activity of NF-κB. Previous studies have shown that Nrf2-dependent HO-1 expression inhibits NF-κB secretion in TNF-α stimulated cell (Pae et al., 2006).

If the skin is constantly exposed to inflammatory stimuli or oxidative stress, skin diseases such as AD will develop. One defense mechanism against this is the upregulation of Nrf2/HO-1 signaling to inhibit the production of adherent molecules, inflammatory cytokines, and chemokines, as well as to inhibit the activation of NF-κB.

Skin barriers that are damaged by external stimuli can cause allergic antigens to penetrate the skin, causing immune reactions and inflammation (Batista et al., 2015). FIL, IVL, and LOR are known to be the major proteins that form epidermal barrier. Production defects and gene mutations in these proteins are important causes of AD pathogenesis. Dysfunction of the skin barrier increases the infiltration of allergens and increases microbial proliferation, thereby increasing the Th2 response of skin tissues (Kim et al., 2008).

Based on these mechanisms, we examined the role of SST in TNF-α/IFN-γ-stimulated HaCaT cells and in DNCB-induced AD-like skin lesions mouse model.

We confirmed that SST increased the expression of HO-1 and the nuclear translocation of Nrf2 and decreased the expression of Keap1 protein in HaCaT cells. Through this, we found that SST up-regulated the Nrf2/HO-1 defense mechanism against oxidative stress. We also observed that SST significantly inhibited the expression of ICAM-1 in HaCaT cells stimulated with TNF-α/IFN-γ and suppressed the binding of THP-1 cells to HaCaT cells. In addition, we examined the expression pattern of ICAM-1 on SST in HO-1-knockdown HaCaT cells. The expression of ICAM-1 gene was increased in HO-1 knockdown cells compared with the cells transfected with control siRNA, increasing the binding of THP-1 cells to HaCaT cells. This suggested that the expression of the ICAM-1 gene was induced by HO-1 gene. We also found that SST reduced NF-κB activity in a dose-dependent manner and enhanced skin barrier function by increasing the expression of skin barrier-forming proteins.

Skin hyperkeratosis, skin inflammation, and skin barrier dysfunction are observed in AD. Therefore, we evaluated those conditions in the visually observable dorsal skin of mice model of DNCB-induced AD-like skin lesions. The results suggested that oral administration of SST significantly alleviated AD-like symptoms by restoring AD severity score, skin thickness, spleen weight, IgE, and IL-4. We also observed the expression patterns of Nrf2, HO-1, NF-κB, and ICAM-1 in mouse dorsal skin tissues. Similar to that observed *in vitro*, the expression of antioxidant-related factors Nrf2 and HO-1 was increased in a dose-dependent manner by oral administration of SST, whereas the expression of inflammatory mediator NF-κB and the adhesion molecule ICAM-1 was decreased in a dose-dependent manner. Furthermore, the level of cytokines was significantly inhibited by oral administration of SST in the dorsal tissue of DNCB-induced mice.

**Figure 10:**
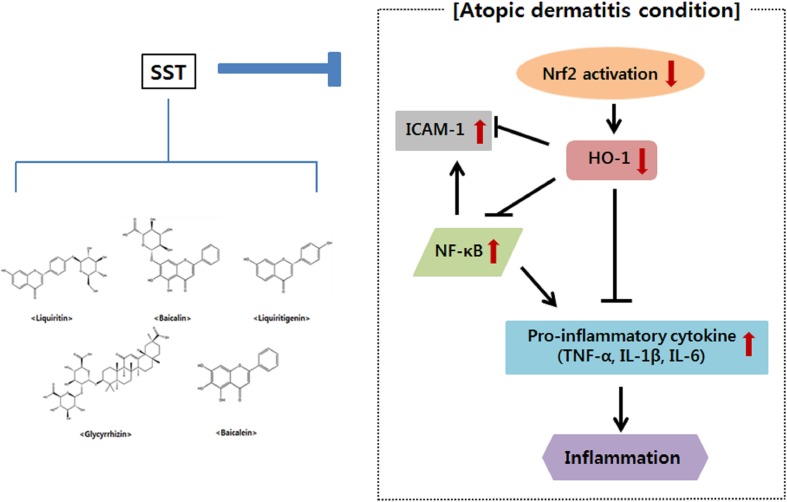
A summary of AD-related genes regulated by SST water extract.

In conclusion, the present study showed that SST inhibited NF-κB activation and ICAM-1 gene expression by up-regulating the Nrf2 and HO-1 genes *in vitro* and *in vivo*, suggesting that SST is associated with a decrease in oxidative stress. SST also increased skin barrier protein and inhibited pro-inflammatory cytokines (**Figure 10**). SST is made up of medicinal herbs needed to prevent and treat chronic inflammatory skin diseases. Therefore, it is assumed that the anti-atopic and anti-inflammatory effects of SST are due to the synergistic effects among the anti-inflammatory components contained in the herbal medicine. Through this, we suggest that SST is a safe traditional herbal medicine for the prevention and treatment of chronic inflammatory skin diseases such as AD.

## Ethics Statement

All animal procedures adhered to the National Institutes of Health guidelines and were approved by the Animal Experiment Ethics Committee of Chonbuk National University (CBNU 2017-0002).

## Author Contributions

MP and D-KK selected the topic. J-HL, BL, and Y-ML performed the experiments. J-HL analyzed the data and wrote the manuscript. All authors have read this manuscript and approved the submission.

## Funding

This work was supported by the National Research Foundation of Korea (NRF) grants funded by the Korean government (MSIP) (2008-0062484) (2015M3A9E3051054).

## Conflict of Interest Statement

The authors declare that the research was conducted in the absence of any commercial or financial relationships that could be construed as a potential conflict of interest.

## Abbreviations

AD, atopic dermatitis; DAPI, 4’,6-diamidino-2-phenylindole dihydrochloride; DMEM, Dulbecco’s modified Eagle’s medium; DNCB, 2,4-dinitrochlorobenzene; FBS, fetal bovine serum; HO-1, heme oxygenase-1; ICAM-1, intercellular adhesion molecule-1; IFN-γ, interferon-γ; IL-1β, interleukin 1-beta; NF-κB, nuclear factor-κB; Nrf2, NF-E2 p45-related factor 2; p-IκBα, phospho-inhibitor of nuclear factor α; SDS-PAGE, sodium dodecyl sulfate-polyacrylamide gel electrophoresis; siRNA, small interfering RNA; SST, *Soshiho-tang*; TBST, Tris-buffered saline containing 0.05% Tween 20; TNF-α, tumor necrosis factor-α.
