# Supplementary material for: Soshiho-Tang, a Traditional Herbal Medicine, Alleviates Atopic Dermatitis Symptoms via Regulation of Inflammatory Mediators
Source: Front Pharmacol. 2019 Jul 3;10:742. doi: 10.3389/fphar.2019.00742 (PMC6626912; doi:10.3389/fphar.2019.00742)
Supplement: Supplementary file 1 [file DataSheet_1.docx]

**Soshiho-Tang, a traditional herbal medicine, alleviates atopic dermatitis symptoms via regulation of inflammatory mediators**

Ji-Hyun Lee, Eun Hee Jo, Bori Lee, Hyeon Min Noh, Sunggu Park, Young-Mi Lee, Dae-Ki Kim^#^, Min Chel Park^##^

Department of Immunology and Institute of Medical Sciences, Medical School, Chonbuk National University, Jeonju, Jeonbuk 54907, Republic of Korea.

^#^ Corresponding author: Dae-Ki Kim, [daekim@jbnu.ac.kr](mailto:daekim@jbnu.ac.kr)

^##^ Corresponding author: Min Cheol Park, spinx11@wonkwang.ac.kr

**
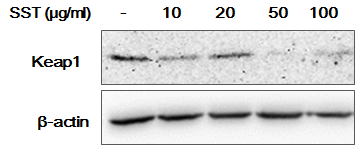
**

**Supplementary Figure 1.** Effects of SST on Keap1 expression. HaCaT cells were treated with various concentrations of SST for 24 h. Keap1 protein levels were detected using western blot.
